# Editorial: Synergistic therapy for invasive fungal infection

**DOI:** 10.3389/fcimb.2023.1227837

**Published:** 2023-07-14

**Authors:** Sunil S. Menghani, Deweshri Nandurkar, Nilesh R. Rarokar

**Affiliations:** ^1^ Department of Pharmaceutical Chemistry, Krupanidhi College of Pharmacy, Bangalore, Karnataka, India; ^2^ Department of Pharmaceutical Chemistry, Dadasaheb Balpande College of Pharmacy, Nagpur, India; ^3^ Computer Aided Drug Design Laboratory, Department of Pharmaceutical Sciences, Mahatma JyotibaFuleyShaikshanikParisar, RashtrasantTukadoji Maharaj Nagpur University, Nagpur, India

**Keywords:** fungal infection, synergistic therapy, fungal resistant, antifungal drugs, amphotericin B

In recent decades, an invasive fungal infection has become a global threat to human health and contributes to 1.7 billion infections and 1.6 million deaths annually. Of note, this epidemiological data is inconsistent due to the lack of criteria for reporting fungal diseases and misdiagnosis problems. Advances in technology and medicine, as well as the use of more aggressive cancer treatments and transplantation for patients with underlying heart, kidney, and liver disease have changed the incidence and treatment of fungal infections. In particular, these advances and successful treatments have significantly increased the risk of fungal infections in people. Thus, the incidence of fungal pathogens has increased dramatically and may contribute to more human morbidity and mortality than is currently believed. Endemic dimorphic fungi such as Aspergillus, Candida, Cryptococcus spp., Pneumocystis jirobesii, and Histoplasma capsulatum and Mucor fungi remain serious commensals. It is the primary fungal pathogen responsible for most cases of fungal disease. Candida albicans is a major cause of mucosal disease, Aspergillus fumigatus is responsible for most allergic fungal diseases, and Trichophyton, especially Rubrum, is responsible for skin infections. Fungal infections is noteworthy in rural areas and thus, becoming a localized problem in tropical and subtropical areas. Subcutaneous fungal infections such as sporotrichosis, chromoblastomycosis, eumycetoma are rampant fungal infection in these area. These fungal infections are invasive in nature as they weaken the host’s immune system. However percentage of prevalence and morbidity rate is more in patients suffering from immunocompromised HIV, Cancers and Tuberculosis, etc.

Azoles, echinocandins, allylamines and polyenes are the four major classes of antifungal agents used to treat candidiasis and other types of fungal infections in humans. Azoles such as fluconazole, itraconazole, posaconazole and voriconazole are considered a first-line drug for the treatment of intractable fungal diseases. Though less toxic but resistance development particularly for candida species limits its use. Resistance associated with polyenes are extremely rarely even on long time prescription but nephrpotoxicity and systemic toxicity have questioned on safety profile. Echinocandins are comparatively safer but again rise in resistance is a severe concern.

A promising strategy to combat resistant microbes is to use combination therapy to extend the longevity and efficacy of current agents. Combining drugs can increase efficacy and specificity and delay the development of resistance compared to single-agent therapy. Moreover, through careful selection of specific drug combinations, microbial drug resistance can not only be neutralized, but even reversed. Combination therapy is already the treatment of choice for many infectious diseases such as HIV, tuberculosis and malaria. As a result, the use of drug combinations to treat fungal pathogens has received a great deal of interest in recent years. Various studies highlight the potential of combination therapy to reverse antibiotic resistance. Combinations of inhibitors have been shown to reverse selective pressure and adversely affect drug-resistant mutants. The nature of drug interactions may change depending on mutation, and drug combinations may act synergistically in resistant mutants. Selection reversal can also occur when resistance to one drug also confers the susceptibility to a second drug, called collateral susceptibility. Treatment of severe invasive fungal infections with two or more antifungal agents has long been a clinical practice. The first application of synergistic therapy in invasive candidiasis is flucytosine and amphotericin B. Flucytosine monotherapy usually causes drug resistance and unanticipated side effects, but amphotericin B alleviates these problems. This combination is recommended by the Infectious Diseases Society of America (IDSA) guidelines for the treatment of candidiasis in patients in certain settings, including those with severe and deep Candida infections that affect the central nervous system (CNS). Moreover, the strong synergistic effect of antivirals and low-dose toxic antifungals allows for more effective control of fungal infections that is more effective yet less toxic than using individual agents.

Synergistic drug combinations have emerged as an effective and practical strategy. Thus combinations therapy shall be incorporated as an integral part of an antifungal and mycosis management program. Furthermore, there is an urgent need to develop new classes of antifungal agents to reduce the death rate date in due of fungal attack.

## Author contributions

SM, contributed in writing the the contents. DN and NR contributed in language editing. All authors contributed to the article and approved the submitted version.

